# Is Dentistry Overcrowded?

**Published:** 1901-04

**Authors:** 


### Editorial.

### IS DENTISTRY OVERCROWDED?

THE approach of the commencement period naturally turns the thoughts of those who expect to graduate in dentistry to the subject of where to locate. To the minority, who expect to follow in the footsteps of their fathers who have preceded them in the profession this may not be important, but to the large majority it is not only a serious problem, but a source of anxiety to themselves and to those directly interested in their future welfare. Possibly no class is more deeply interested in this than those who have the training of these young men. The teachers feel that they are in large degree responsible for the success or non-success of the student, and would gladly point out the way to overcome at least a portion of the initial struggle which is a part of the experience of every young life. While wealth may change the character of that struggle, there is always something to be overcome, something to be learned, something which money cannot buy, which must be ingathered whether it be for good or evil, for the advancement of the man to higher things or for his debasement.

The strenuous character of our every-day life, the competitions and the increasing difficulty of securing more than a mere living in the various subordinate positions, is rapidly tending to a condition of affairs the despair of the political economist. The hopelessness of ever securing an independent position in the ordinary occupations of men is fast driving young men of limited means into the professions. This tendency has long since been noted, and it is to this that is largely due the counter-efforts of those already in the professional ranks to drive back the ever-increasing flood. To effect this all sorts of legal obstructions have been placed in the way of the young graduate. These legal forms have another meaning to the member of a State board, or, at least, he tries to convince himself that he is working for the elevation of his profession, and some may even succeed in forcing themselves to believe it, but underlying all this effort is the one paramount feeling that the profession is already overcrowded and the new men are not wanted. And further than that, the colleges are doing a great wrong in that they fail to discourage young men from taking up the study of dentistry.

This question of overcrowding has been incidentally alluded to heretofore in these editorial pages, but a more extended examination of the subject seems not only important at this period, but absolutely necessary, that this ever-recurring charge be met with facts from reliable sources, that demonstrate beyond mere words that this country is not only not overcrowded with dentists, but cannot possibly reach that condition for many years in the future.

It must be clear to all minds that overcrowding means more dentists than the population will support. If, then, it can be shown that the number of dentists bears no proportion to the number that could be comfortably supported, the senseless cry should not be heard for some generations to come.

The writer has endeavored to meet this demand by comparing the number of dentists with population in several of our large cities, smaller towns, and villages, and the possible number that may be claimed as rightfully belonging to the practitioner, or from which he may expect to secure a clientele.

Beginning with New York City, and taking the number of dentists from Polk's *Dental Register* and the population from recent census returns, it is shown that with a population of 3,437,202, New York City contains 1143 dentists. This means New York City proper, Brooklyn, and adjacent boroughs. This gives one dentist to 3007 people.

Chicago has a population of 1,698,575. Number of dentists 1025, or one dentist to 1657 people.

Philadelphia, the third city in size, with a population of 1,293,-697, has 664 dentists, or one dentist to 1948 persons. This city has probably 1400 dental students who may properly be regarded as practitioners. This number added to 664 equals 2064, or one dentist to 627.

This may be applied, with proportionate degree, to all cities having dental colleges. The writer has, however, not considered it in those cities most deeply affected,—Chicago, New York, Boston, Baltimore, and Cincinnati.

Boston has 560,892 inhabitants and 462 dentists, or one to 1214 people.

Baltimore numbers 508,957, with 254 dentists, or one to 2004.

Cincinnati has 325,902 people and 217 dentists, or one to 1502.

Richmond, Va., has a population of 85,050, with 51 dentists, or one dentist to 1667.

New Orleans, with a population of 287,104, has 119 dentists, or one dentist to 2412.

Omaha, Neb., numbers 102,555, with 55 dentists, or one dentist to 1864.

Kansas City, Mo., has 163,752, with 155 dentists, or one to 1057.

San Francisco, with a population numbering 342,742, has 425 dentists, or one to 806.

Portland, Me., with 90,426 people, supports 68 dentists, or one to 1335.

Minneapolis, with 202,718, has 100 dentists, or one dentist to 2027.

Albany, N. Y., has a population of 94,151, with 35 dentists, or one to 2690.

In the smaller towns, numbering some 25,000 people, two are selected: New Britain, Conn., population 25,998, with 9 dentists, or one dentist to 2888. Cedar Rapids, Iowa, population 25,656, with 22 dentists, or one to 1166.

Villages support rather more than their share of the dental fraternity. Pen Yan, N. Y., with a population of 5050, has 5 dentists, or one to 1010 people. Griffin, Ga., with a population of 5000, has 6 dentists, or one to 833.

To arrive at some definite conclusion in regard to the value of these statistics, it must be borne in mind that the maximum number of patients that can properly be waited upon per day of eight hours will not exceed six. This does not include examination cases. This will give eighteen hundred for a year of three hundred working days. It must be remembered, however, that each patient will require several sittings, and averaging this at four for a person, it will make 450 as the actual clientele of the dentist. If this be made, in round numbers, 500, and allow 30,000 dentists for the United States (Polk makes it 26,500), there will be 15,000,000 people treated in dental offices yearly. The population of the entire country is 76,000,000, which leaves 61,000,000 unprovided with dental aid. Making due allowance for the poorer classes, blacks, and Indians, there will still be a large population, probably at least 30,000,000, that should require the services of the dental profession.

It will be observed, in analyzing the figures previously given, that not one of the larger cities is as yet overcrowded, not even Philadelphia, which comes the nearest to the minimum limit.

Some of the results of this examination present peculiar features. For instance, New York City would seem, with its one dentist to three thousand inhabitants, to be a remarkably good locality for a young man to settle in. It must, however, be remembered that New York has a very large foreign population, and perhaps more than any other city of that kind of population that gives no attention to sanitary conditions, and least of all to the care of the oral cavity. On the other hand, the attractions of a great metropolis draw people from all sections of the country and the world, thus largely increasing the possible number to draw upon.

San Francisco would seem, with its one dentist to eight hundred and six people, to be very much overcrowded as compared with other cities, and yet it has not reached the minimum previously stated.

With this single exception there is no city in this country where the young dentist need to hesitate in regard to settlement. There is apparently room for more everywhere.

The present April will find a large number who will pass out from the dental colleges of the United States and Canada. Before these come to any decision upon the locality in which to settle they should carefully study the statistics as given in the *Dental Register,* to which reference has been made. While this directory may not be absolutely correct, it is the nearest to reaching that desirable result of any heretofore published, and it will give a very definite idea of future possibilities for the young practitioner.

The graduate should take time, months if necessary, and having selected a favorable location, settle and *remain*. Unless capital is abundant, avoid all large cities. The struggle is here too severe without good financial support to enable the beginner to wait for the practice that will come in time.

